# Could Fingolimod Combined with Bevacizumab Be a New Hope in Glioblastoma Treatment?

**DOI:** 10.3390/cimb47060394

**Published:** 2025-05-26

**Authors:** Murat Baloglu, Canan Vejselova Sezer, Hüseyin Izgördü, Ibrahim Yilmaz, Hatice Mehtap Kutlu

**Affiliations:** 1 Department of Neurosurgery, Eskisehir City Hospital, Ministry of Health, Eskisehir 26080, Turkey; 2 Department of Biology, Faculty of Arts and Sciences, Kutahya Dumlupinar University, Kutahya 43100, Turkey; cananveyselova@gmail.com; 3 Lung and Pleural Diseases Application Center, Faculty of Medicine, Eskisehir Osmangazi University, Eskisehir 26040, Turkey; huseyinizgordu@gmail.com; 4 Department of Pharmacovigilance, Doctor Ismail Fehmi Cumalioglu City Hospital, Ministry of Health, Tekirdag 59020, Turkey; dryilmazi@yahoo.com; 5 Department of Medical Services and Techniques, Vocational School of Health Services, Istanbul Rumeli University, Istanbul 34570, Turkey; 6 Department of Biology, Faculty of Science, Eskisehir Technical University, Eskisehir 26555, Turkey; hmkutlu@eskisehir.edu.tr

**Keywords:** apoptosis, bevacizumab, fingolimod, glioblastoma

## Abstract

Glioblastoma, classified as a grade IV astrocytoma, is an aggressive and malignant primary brain tumor with no known cure. Despite the implementation of standard medical and surgical treatment protocols, the disease often progresses with unsatisfactory outcomes. This study aimed to evaluate the cytotoxic, proapoptotic, and antimetastatic effects of anti-angiogenic monoclonal antibody bevacizumab combined with the sphingosine-1-phosphate receptor modulator fingolimod on rat glioma C6 cells. The cytotoxicity of bevacizumab and fingolimod was evaluated using the MTT assay. Proapoptotic activity was assessed through flow cytometric analyses, including Annexin V–FITC staining, caspase 3/7 activation, and mitochondrial membrane potential measurements. Morphological changes were examined using confocal microscopy. Antimetastatic effects were evaluated via anti-migration and colony formation assays. The combination of bevacizumab and fingolimod exhibited antiproliferative, cytotoxic, proapoptotic, and antimetastatic effects on C6 glioma cells at low IC_50_ concentrations. Based on growth inhibitory, proapoptotic, and antimetastatic activities on C6 glioma cells, the combination of bevacizumab and fingolimod demonstrates significant growth-inhibitory, proapoptotic, and antimetastatic activities against C6 glioma cells, suggesting its potential as a promising pharmacotherapeutic approach for the treatment of glioblastoma.

## 1. Introduction

Glioblastoma, classified as a grade IV astrocytoma, is the most common and aggressive type of primary brain tumor. It is highly invasive, associated with high mortality rates, and accounts for approximately 60% of malignant brain tumors [1]. Despite advances in treatment strategies, including extensive surgical resection, radiotherapy, and chemotherapy, glioblastoma remains largely refractory to therapy, with poor patient outcomes. Epidemiological data indicate a median overall survival of approximately 14 months, with a two-year survival rate of less than 5% [2]. Approximately 85% of these tumors exhibit a highly invasive nature, predominantly originating from the temporal and frontal lobes of the brain [3]. More than half of glioblastoma cases (approximately 60%) result in death within one year of diagnosis [4]. Without treatment, the average survival time is approximately 3 months, while standard clinical treatments extend survival to an average of 14–21 months [5]. Although five-year survival rates have shown slight improvement over the past two decades, the prognosis for glioblastoma remains extremely poor, with no curative treatment available [4].

Although it is less common than breast, lung, and colorectal cancers, the number of deaths attributed to glioblastoma has been steadily increasing over the years [6,7]. The prognosis for patients diagnosed with glioblastoma remains dismal, with survival times significantly limited. This underscores an urgent need for pharmacological agents capable of providing effective treatment for glioblastoma, a disease that not only causes substantial loss of life but also imposes considerable financial burdens on healthcare systems. Currently, there is no effective drug or surgical treatment modality for glioblastoma [8]. Surgical resection, a cornerstone of cancer treatment, is generally ineffective for glioblastoma due to the inability to achieve clear surgical margins. The aggressive biological characteristics of glioblastoma, coupled with the complexities of brain anatomy, often preclude gross total resection. Glioblastoma spreads micro invasively into healthy brain tissues, making it difficult to distinguish between tumor and non-tumor areas during surgery.

Consequently, residual cancer cells left behind post-surgery contribute to a high rate of tumor recurrence. The challenges of surgical resection are further compounded when glioblastoma is located in critical functional areas of the brain, such as the motor cortex or speech centers. In such cases, complete tumor removal may not be feasible, as it risks causing severe neurological impairments. Additionally, glioblastomas situated in deep brain structures, such as the basal ganglia and brainstem, are often inaccessible surgically, and attempts at resection in these areas may result in increased neurological complications. Glioblastoma is a genetically and biologically heterogeneous tumor, and residual cells left behind after surgical resection can contribute to rapid tumor regrowth, even when a substantial portion of the tumor appears to have been removed. Post-surgical complications, such as brain edema or bleeding, may further limit the success of surgical interventions. Additionally, microscopic tumor cells that cannot be removed during surgery proliferate quickly, leading to recurrence within a short time frame.

Treatment challenges extend beyond surgery. The blood–brain barrier poses a significant obstacle, preventing many therapeutic agents from effectively reaching the brain tissue and, consequently, the tumor. Even in cases where drugs successfully cross the BBB, resistance to treatment often develops. Factors such as molecular heterogeneity and clonal evolution within the tumor further reduce the efficacy of pharmacological interventions. The hypoxic and acidic tumor microenvironment created by glioblastoma can also contribute to mechanical resistance, diminishing drug effectiveness.

On a molecular level, glioblastoma exhibits frequent overactivation of growth factors—such as epidermal growth factor receptor (EGFR), platelet-derived growth factor (PDGF), and vascular endothelial growth factor (VEGF)—and associated signaling pathways, including phosphatidylinositol 3-kinases (PI3K)/protein kinase B (AKT)/mammalian target of rapamycin (mTOR), and mitogen-activated protein kinase (MAPK). Furthermore, glioblastoma often establishes an immunosuppressive microenvironment that impairs the immune system’s ability to combat the tumor. This suppression is exacerbated by the overexpression of immune checkpoint proteins, such as programmed death ligand 1 (PD-L1), which inhibit the immune response. Multidrug resistance mechanisms within glioblastoma cells also contribute to reduced treatment effectiveness. Since glioblastoma drugs are generally not target-specific, they may produce undesirable adverse/side effects in healthy cerebral tissues. VEGFs, particularly VEGF-A, have been identified as key players in triggering angiogenesis in tumors. They initiate endothelial cell signaling by affecting receptor tyrosine kinase enzymes [9]. VEGF can promote cell growth and survival through these pathways and can increase vascular permeability. Furthermore, VEGFs contribute to the metabolic needs of tumors during growth. Angiogenesis is a critical process in tumor progression. Recently, pharmacological research on drug design has prioritized VEGF-A due to its potential as an intracellular therapeutic target for limiting angiogenesis. Additionally, it holds great potential for minimizing and normalizing tumor vasculature [10]. In vivo investigations in multiple tumor models have proven the validity of this technique, demonstrating that angiogenesis suppression using a VEGF monoclonal antibody reduces tumor growth [9].

Bevacizumab, an antibody that targets VEGF-A, is among the initial targeted therapies for inhibiting angiogenesis. Marking the beginning of a new line of anticancer medications, bevacizumab remains a well-studied anti-angiogenic therapeutic agent. Initially approved for colorectal cancer treatment, it has since been used for breast cancer, glioblastoma, ovarian cancer, and other malignancies [11]. Fingolimod, a structural sphingosine and an immunomodulator, is used for the treatment of multiple sclerosis. It is derived from the fungal metabolite myriocin [12]. This immunomodulator agent has also been found effective in the treatment of various cancer types [13]. However, the mechanisms by which it exerts anticancer activity on glioma cells are not yet fully understood. Sphingosine kinase phosphorylates this immunomodulatory agent, which functions as an agonist of the sphingosine-1-phosphate receptor. The drug, also known as FTY720-P, is a cell-permeable aminopropanediol immunosuppressant that binds to four of the five G protein-coupled S1P receptors (S1P1, S1P3–5), activating downstream pathways through G protein isoform signaling (Gs, Gi, Gq, and G12/13) [14]. These pathways frequently regulate several biological processes, including cell proliferation and survival [15]. Recent research has also revealed that FTY720 can exert receptor-independent epigenetic effects mediated by intracellular signaling and histone deacetylases [16]. The intracellular targets of FTY720 inhibition include sphingosine kinase 1, cell transporters, autotaxin, cell migration mediators such as E-cadherin, N-cadherin, and vimentin, sphingosine-1-phosphate receptor 1, and cell cycle regulators such as cyclin D1, CDK2, and CDK4 [15]. Additionally, it influences PI3K/Akt pathway proteins 14-3-3, cytochrome c, and caspases [17].

To date, the effects of bevacizumab alone in the treatment of glioblastoma have been extensively studied. However, studies investigating fingolimod as a treatment for glioblastoma are sparse. Furthermore, no high-evidence studies have been identified that examine the combined effects of these two agents against glioblastoma. This research aims to address the efficacy of bevacizumab and fingolimod combination on propagating the death of glioma cells. Specifically, the cytotoxic, antiproliferative, proapoptotic, and antimetastatic effects of these agents in combination were tested in vitro.

## 2. Materials and Methods

### 2.1. Materials

Dimethyl sulfoxide (DMSO; Cat. No. 67-68-5) was supplied by Sigma-Aldrich (St. Louis, MO, USA). Fetal bovine serum (FBS; Cat. No. 1943609-65-1), penicillin–streptomycin (PS; Cat. No. 516106), Dulbecco’s Modified Eagle’s Medium (DMEM; Cat. No. D5030), and Dulbecco’s Phosphate-Buffered Saline (PBS, pH 7.4; Cat. No. 806552) were obtained from GIBCO (Grand Island, NE, USA).

### 2.2. Culture of C6 Cells

C6 (CLL-107), a rat glioma cell line, was obtained from the American Type Culture Collection. For cell culture, 10% FBS and 1% PS were added to DMEM. The cells were maintained under standard culture conditions at 37 °C, 5% CO_2_, and high humidity in a Thermo Scientific Heracell 150i incubator (Thermo Fisher Scientific, Waltham, MA, USA) Proliferating cells were passaged twice a week, and all experiments utilized flasks with 85% confluency [18].

### 2.3. Preparation and Applications of Drugs to C6 Cells

C6 cells (3 × 10^4^ cells/well) were seeded into 24-well plates containing DMEM and incubated for 48 h. Following incubation, the cells were treated with bevacizumab (Avastin^®^ 25 mg/mL, Genentech, Inc., South San Francisco, CA, USA) and/or fingolimod (100 µM; Cas. No. 162359-56-0, Sigma-Aldrich, St. Louis, MO, USA) (Table 1).

### 2.4. MTT Colorimetric Assay

The cytotoxicity of bevacizumab and fingolimod was assessed using a commercial 3-(4,5-dimethylthiazol-2-yl)-2,5-diphenyltetrazolium bromide (MTT) assay. C6 cells were seeded in 96-well plates at a density of 5 × 10^3^ cells/well and treated separately with various concentrations of bevacizumab and fingolimod for 24 and 48 h. Following incubation, 20 µL of MTT solution was added to each well, and the plates were incubated for an additional 4 h. Afterward, the medium in each well was replaced with 200 µL of dimethyl sulfoxide (DMSO), and absorbance was measured using an ELISA reader (Bio-Tek HTx Synergy, Winooski, VT, USA) [19]. The IC_50_ values were calculated from the obtained viabilities and the combination of IC_50_ values was used for all other experimentations. The cytotoxicity type was determined by the formula of combination index (CI; CI = d1/D1 + d2/D2 were CI < 1 indicates synergism, CI = 1 additive cytotoxicity, CI > 1 antagonism [20].

### 2.5. Annexin V–FITC Staining

Annexin V–FITC staining was performed to assess the apoptotic potential of the drugs on C6 cells. This method utilizes Annexin V–FITC, which binds specifically to phosphatidylserine exposed on the cell surface, making it an effective marker for detecting early apoptotic events before the loss of membrane integrity. C6 cells (5 × 10^5^ cells/well) were treated with the combination of IC_50_ values of bevacizumab (0.06 µM) and fingolimod (8.27 µM) in 6-well plates for 24 h at 37 °C under standard cell culture conditions, with all experiments performed in triplicate. Following incubation, cells were trypsinized, washed with PBS, and resuspended in separate tubes (1 × 10^3^ cells per tube). Then, 100 µL of cell suspension was mixed with 100 µL of Annexin V–FITC reagent and incubated for 15 min at room temperature in the dark. After staining, cells were analyzed according to the manufacturer’s instructions using the Muse™ Cell Analyzer (Merck Millipore, Hayward, CA, USA).

### 2.6. Caspase-3/7 Activity Analysis

Effector caspases 3 and 7 play a central role in apoptosis by degrading cellular substrates and orchestrating controlled cell lysis. Caspase-3/7 activation is a hallmark of apoptosis, occurring downstream of both intrinsic (mitochondrial) and extrinsic (death receptor-mediated) apoptotic pathways.

C6 cells were treated with a combination of IC_50_ values of bevacizumab (0.06 µM) and fingolimod (8.27 µM) in 6-well plates for 24 h, in triplicate. After incubation, 50 μL of cell suspension was mixed with 5 μL of Muse^®^ caspase-3/7 working solution (prepared at a 1:8 ratio in 1× PBS). For flow cytometry analysis, 7-aminoactinomycin D (7-AAD), a DNA-binding fluorescent intercalator, was used. A working solution was prepared by mixing 2 µL of 7-AAD with 148 µL of 1× assay buffer. Then, 150 µL of the 7-AAD working solution was added to each sample, and the samples were incubated according to the manufacturer’s protocol. Finally, all samples were analyzed using the Muse™ Cell Analyzer (Merck Millipore, Hayward, CA, USA).

### 2.7. Mitochondrial Membrane Potential Analysis

Mitochondrial membrane potential was evaluated as an indicator of mitochondrial toxicity and/or oxidative stress induced by the applied drugs. The assay was performed in triplicate using the Muse MitoPotential Kit (Luminex, Wegberg, Germany) following the manufacturer’s instructions. Control and test C6 cells were exposed to the combination of IC_50_ values of bevacizumab (0.06 µM) and fingolimod (8.27 µM) in 6-well plates for 24 h, harvested with trypsin, and stained with the working solution for 20 min followed by 7-AAD staining for 5 min at 37 °C. The samples were analyzed using the Muse Cell Analyzer (Millipore, Schwalbach, Germany) [21].

### 2.8. Colony Formation Assay

The colony formation assay was conducted to evaluate the colony inhibition effects of combination of IC_50_ values of bevacizumab (0.06 µM) and fingolimod (8.27 µM), serving as an indicator of the antimetastatic potency of the tested agents. C6 control and test cells (1 × 10^3^ cells/well) were seeded into six-well culture plates in triplicate and incubated for 14 days. Following incubation, the cells were stained with MTT (5 mg/µL in PBS) for 60 min, and colonies were counted manually. The colony formation rate was calculated as the ratio of the number of colonies to the number of seeded cells. This method was adapted from Du et al. [22].

### 2.9. Migration Assay

The migration assay was conducted to evaluate the indirect effects of bevacizumab and fingolimod on cellular adhesion molecules, serving as an indicator of their ability to inhibit migration in C6 cells. C6 cells were seeded in six-well plates in triplicate and allowed to reach confluency. An artificial scratch was created in each well using the tip of a sterile pipette. The wells were then washed with PBS, and initial images of the scratches were captured under a light microscope to document the starting point. Test groups of C6 cells were treated with the combination of IC_50_ values of bevacizumab (0.06 µM) and fingolimod (8.27 µM) for 24 h, while control groups were incubated with fresh medium under identical conditions. After the incubation period, the closure of the artificial scratches was examined under a microscope, and images were captured to assess the migration activity of the C6 cells. This method was adapted from Wang et al. [17].

### 2.10. Evaluation of Morphological Changes

Morphological changes in C6 cells were evaluated to observe cellular alterations induced by the treatment. C6 cells (3 × 10^5^ cells/well) were seeded on sterilized coverslips in six-well plates in triplicate and treated with the combination of IC_50_ values of bevacizumab (0.06 µM) and fingolimod (8.27 µM) for 24 h under standard cell culture conditions (37 °C, 5% CO_2_). Untreated cells served as controls and were incubated with fresh medium for the same duration. At the end of the incubation period, all samples were washed with PBS and stained with phalloidin and acridine orange for 20 min at room temperature in the dark. The samples were then imaged using a fluorescent microscope (Leica DM 6000B, Amsterdam, The Netherlands) [23].

### 2.11. Statistical Analysis

Analysis of the data was performed using one-way analysis of variance (ANOVA) followed by Tukey’s honestly significant difference (HSD) post-test, using GraphPad Prism for Windows (Version 8.0, GraphPad Software Inc., San Diego, CA, USA). A *p*-value of <0.05 was considered statistically significant.

## 3. Results

### 3.1. MTT Assay

**Figure 1:**
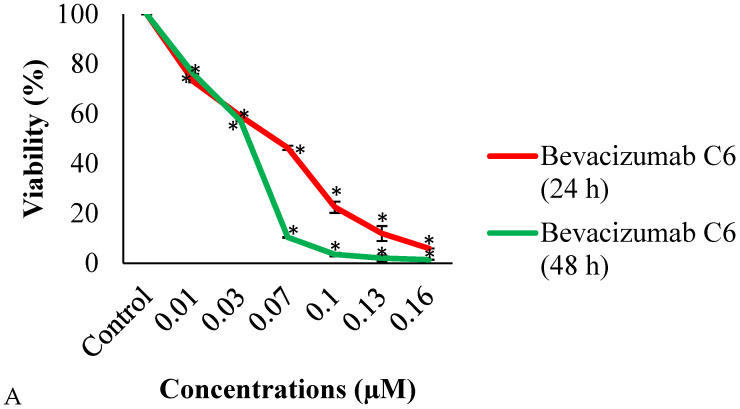

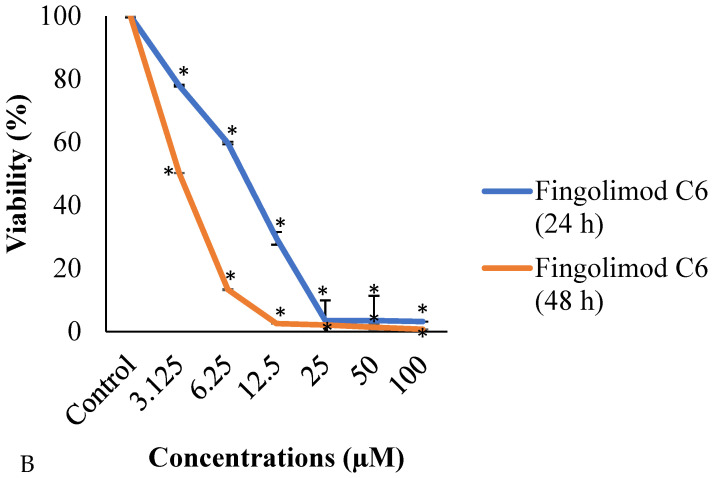
Viability of C6 cells treated with bevacizumab (**A**) and fingolimod (**B**) for 24 and 48 h. * (*p* < 0.05).

The assay was performed to detect the cytotoxicity and dose–response effect of bevacizumab and fingolimod on C6 cells. It was found that cell viability decreased in different bevacizumab concentrations applied to C6 glioma cells at 24 and 48 h of exposure. Cell viability decreased as the bevacizumab concentration increased at both 24 and 48 h of exposure. While the highest growth inhibition was observed at the highest bevacizumab concentrations to which C6 cells were exposed, the viability decrease was found to be statistically significant (*p* < 0.05) after 24 and 48 h of incubation at all concentrations (Figure 1A).

The IC_50_ values of bevacizumab in C6 glioma cells were 0.06 μM at 24 h and 0.036 μM at 48 h. The IC_50_ values of fingolimod in C6 cells were 8.27 μM at 24 h and 3.14 μM at 48 h.

**Figure 2:**
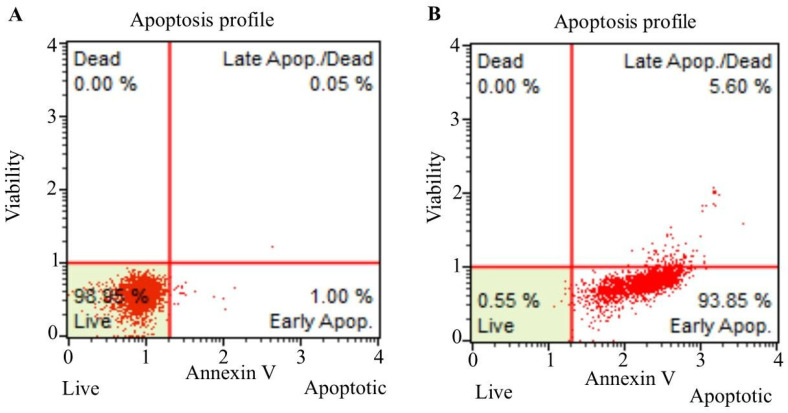
Apoptosis profiles of C6 control cells (**A**) and C6 cells treated with the combination of bevacizumab and fingolimod (**B**) for 24 h.

**Figure 3:**
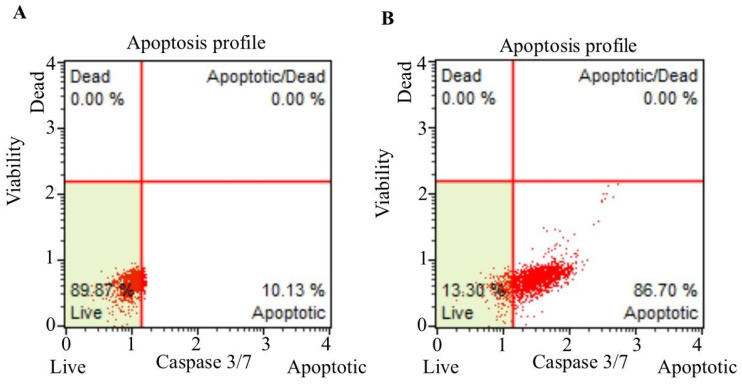
Caspase-3/7 staining profiles of C6 control cells (**A**) and C6 cells treated with the combination bevacizumab and fingolimod (**B**) for 24 h.

**Figure 4:**
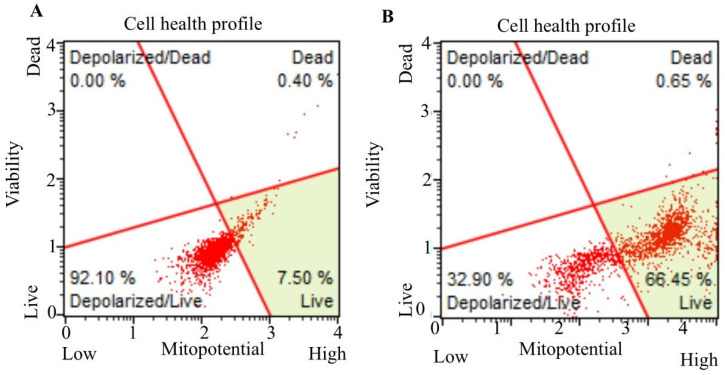
Cell health profiles of C6 control cells (**A**) and combination of bevacizumab- and fingolimod-treated C6 cells (**B**) for 24 h.

**Figure 5:**
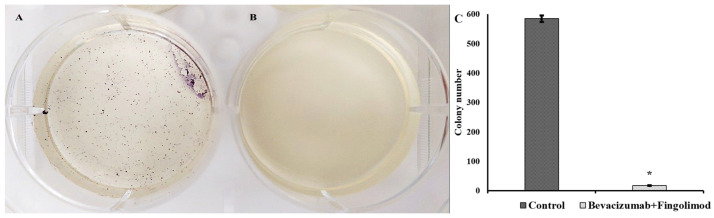
Inhibitory effects of bevacizumab plus fingolimod on C6 cells: (**A**) control group, (**B**) test group, and (**C**) comparison of statistical significances of colony numbers between the groups. * (*p* < 0.05 compared to control cells).

**Figure 6:**
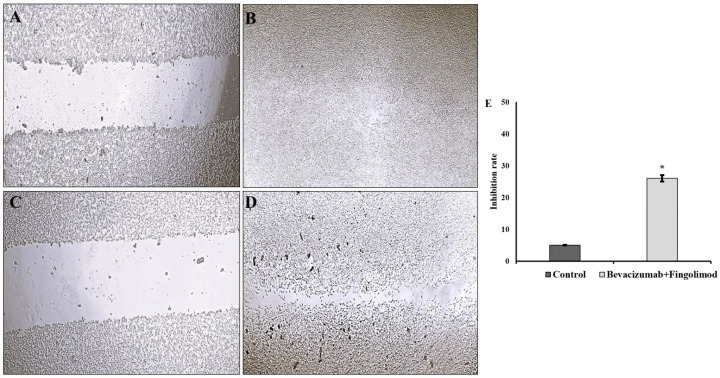
Light microscopy images of C6 cells (10×). (**A**) Control cells at 0 h; (**B**) control cells at 24 h; (**C**) C6 cells exposed to bevacizumab plus fingolimod at 0 h; (**D**) C6 cells exposed to bevacizumab/fingolimod at 24 h; (**E**) comparison of statistical significance of inhibition rate between the groups. * (*p* < 0.05 compared to control cells).

**Figure 7:**
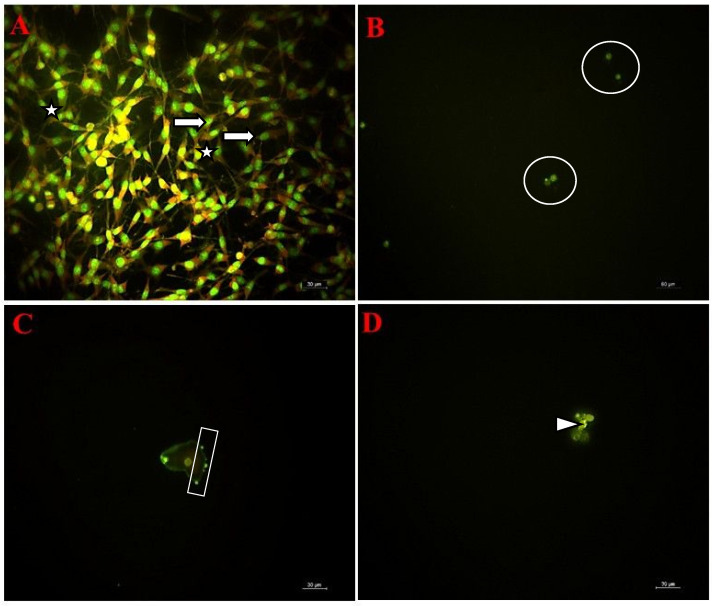
Fluorescent microscopy micrographs of C6 cells (20×). (**A**) Control cells: arrow-compact nuclei, asterisk-compact cytoskeleton; (**B**–**D**) cells exposed to bevacizumab plus fingolimod combination for 24 h: circle-shrunk cells with circular morphology, arrowhead-chromatin condensation, and rectangle-blebbings on membrane.

Fingolimod caused cytotoxicity in C6 cells after 24 and 48 h of incubation. It was found that viability decreased as the applied concentrations increased. The highest decrease in viability was observed at the highest concentrations of 100, 50, and 25 μM. Fingolimod significantly (*p* < 0.05) decreased the viability of C6 cells at all treatment doses at both 24 and 48 h (Figure 1B). The combination of IC_50_ values of the agents calculated from the MTT results were applied to the cells in all experiments (Table 1, Figure 2, Figure 3, Figure 4, Figure 5, Figure 6 and Figure 7). The combination index (CI) value for the bevacizumab and fingolimod combination at 24 h was calculated as CI = 1 that indicates the additive cytotoxicity caused by combined bevacizumab and fingolimod exposure.

### 3.2. Annexin V–FITC Analysis

In order to determine the death type of C6 cells triggered by bevacizumab and fingolimod, the Annexin V assay was performed. Flow cytometry analysis of C6 control cells after annexin V staining showed that the percentage of viable cells was 98.95%. In this group, the early apoptotic cells were 1.00% and the late apoptotic cells were 0.05%. In the C6 control group, the total apoptotic cells were 1.05% (Figure 3A). The data obtained after the bevacizumab/fingolimod treatment of C6 cells showed that the percentage of viable cells was 0.55%. In this group, early apoptotic cells were 93.85%, late apoptotic cells were 5.60%, and total apoptotic cell percentage was 99.45% (Figure 3B).

### 3.3. Caspase-3/7 Staining

The caspase-3/7 staining was performed to determine the caspase-dependence of apoptosis induced by fingolimod and Bevacizumab on C6 cells. The staining profile of the C6 control cells revealed that 89.87% of the cells were viable, while 10.13% were apoptotic (Figure 3A). In contrast, the bevacizumab- and fingolimod-treated C6 cells exhibited 13.30% viability and 86.70% apoptosis, indicating a significant increase in the apoptotic cell percentage (Figure 3B).

### 3.4. Mitochondrial Membrane Potential Measurement

The mitochondrial dependency/independency apoptosis was tested, caused by fingolimod and bevacizumab on C6 cells, by the measurement of changes in the mitochondrial membrane potential. Flow cytometry analysis revealed that untreated C6 cells displayed a cell health profile consisting of 7.50% live cells, 92.10% depolarized/live cells, and 0.40% dead cells. The total depolarized C6 control cell percentage was found to be 92.10% (Figure 4A). In contrast, C6 cells treated with the bevacizumab and fingolimod combination for 24 h showed a cell health profile of 66.45% live cells, 32.90% depolarized/live cells, and 0.65% dead cells. Notably, the percentage of depolarized cells after combination treatment was reduced to 32.90% (Figure 4B).

### 3.5. Colony Formation Assay Results

The potency of bevacizumab and fingolimod on the inhibition of tumor growth was tested by colony formation assay. The results indicated that the colony number of C6 control cells was 584 (±11) after the incubation period (Figure 5A). In the C6 cells exposed to bevacizumab plus fingolimod, no colony formation was detected except for small colonies (~17 ± 1) that were observed under a microscope (Figure 5B).

This implies a strong (*p* < 0.05) (Figure 5C) inhibitory effect of bevacizumab plus fingolimod on the colony formation of C6 glioma cells as a sign of antimetastatic effect.

### 3.6. Migration Assay Results

The antimetastatic capacity of bevacizumab and fingolimod on C6 cells was tested by migration assay. The results revealed that C6 cells maintained their normal proliferation feature after 24 h of incubation with fresh culture medium. The initial artificial scratch (1.2 ± 0.1 mm) was nearly closed (0.06 ± 0.002 mm) at the end of the incubation period (Figure 6A,B). The bevacizumab–fingolimod combination inhibited the migration capability of C6 cells (initial artificial scratch area 1.3 ± 0.3 mm) after exposure for 24 h (Figure 6C,D). It is shown in Figure 6D that the artificial scar area is still open (0.33 ± 0.01 mm final scratch area), which implies the inhibitory effects of the agent combination.

### 3.7. Fluorescent Microscopy Findings

The morphological changes in C6 cells caused by fingolimod and bevacizumab were evaluated by the fluorescent microscopy technique. The C6 control cells, incubated in fresh medium for 24 h, displayed a compact morphology with intact nuclei and cell structures. The cells maintained a fusiform shape, and the number of adherent cells was notably high (Figure 7A).

In contrast, treatment with the bevacizumab and fingolimod combination led to a marked decrease in the confluency of adherent cells. Treated C6 cells exhibited a shrunk, circular morphology compared to the control cells (Figure 7B). Additional morphological changes, including chromatin condensation and membrane blebbing, were observed (Figure 7C,D).

## 4. Discussion

Glioblastoma is an aggressive and lethal primary brain tumor of the central nervous system. It is characterized by high invasiveness and resistance to treatment, resulting in rapid disease progression and low survival rates among patients. Despite many pharmacological studies and major advances in pharmaceutical technology, glioblastoma remains an incurable tumor [24,25].

Combining drugs in glioblastoma treatment has emerged as a promising approach, enabling the simultaneous inhibition of multiple molecular pathways while addressing tumor heterogeneity. This strategy aims to enhance treatment efficacy by reducing both tumor growth and drug resistance [26]. In this study, we investigated the anticancer potential of a combination of bevacizumab and fingolimod in C6 rat glioma cells, providing insights into their possible role in glioblastoma therapy. Despite recent advances in treatment modalities, glioblastoma remains a fatal disease. This research evaluates whether these agents, used in combination, could offer a therapeutic advantage.

Currently, the standard treatment for glioblastoma typically involves maximal safe surgical resection followed by radiotherapy and chemotherapy with temozolomide. Additional therapies, including tumor-treating fields, carmustine wafers, and bevacizumab, are commonly employed as adjuvant treatments to the primary therapeutic regimen [27].

Fingolimod, a sphingosine-1-phosphate receptor modulator, has been reported to exhibit anticancer effects. The therapeutic potential of fingolimod in rat C6 glioma cells has also been investigated and has been reported that fingolimod significantly reduces the survival rate and colony formation of C6 cells while delaying gap closure in migration assays [28,29,30].

The cytotoxicity study performed in this research revealed that the combination of bevacizumab and fingolimod exerted concentration- and time-dependent cytotoxic effects on C6 cells at 24 and 48 h of treatment. Both agents caused statistically significant (*p* < 0.05) reductions in cell viability at all tested concentrations.

Consistent with these findings, prior studies have reported unexpected response rates to bevacizumab in glioma treatment. For instance, the AVF3708g trial demonstrated that bevacizumab prolonged progression-free survival in glioblastoma patients compared to controls. The mean progression-free survival was reported as 5.6 months for irinotecan and bevacizumab combined and 4.2 months for bevacizumab alone [30]. Additionally, a phase 3 study found that bevacizumab, in combination with lomustine, significantly reduced the risk of disease progression [30]. These findings underscore the potential benefits of using bevacizumab in combination therapies, including improvements in progression-free survival and overall survival in glioblastoma patients [31,32].

In the treatment of glioblastoma, the AvaGlio/BO21990 study reported a 36% reduction in cancer progression with bevacizumab exposure. Furthermore, the combination of bevacizumab with radiotherapy and temozolomide demonstrated improved outcomes compared to radiotherapy and temozolomide alone. Similar findings were observed in the RTOG0825 study [33,34].

Unlike therapies that directly target cancer cells, bevacizumab targets cancer cells, normal cells, and the extracellular tumor microenvironment. This complexity means that the effects of VEGF inhibition are often tumor- and microenvironment-specific. Despite advances in understanding the mechanisms of resistance to anti-angiogenic therapy, clinical strategies to overcome bevacizumab resistance remain inadequate [11]. Given this resistance challenge, this study investigated the combination of bevacizumab and fingolimod in rat glioma cells. The results demonstrated that the combination effectively induced apoptosis by activating caspases and altering mitochondrial membrane potential. The proapoptotic efficacy observed here aligns with the potential benefits of combining immunomodulators like fingolimod with bevacizumab, as highlighted in the literature. Consequently, bevacizumab remains a key agent in the treatment of cancer patients [11].

Fingolimod, meanwhile, has emerged as a promising therapeutic agent in many types of cancer; however, its effect on glioblastoma has been addressed in a very limited number of studies. In the present study, the proapoptotic effects of fingolimod in combination with bevacizumab in C6 cells were demonstrated using both flow cytometry and fluorescent microscopy. The morphological changes detected in glioma cells were considered to be typical apoptosis indicators and consistent with the flow cytometry findings. In addition, in this study, it was found that the combination of bevacizumab and fingolimod suppressed migration in C6 cells in short-term treatments and prevented colony formation in long-term treatments. These findings align with previous reports showing that fingolimod (FTY720) significantly improved treatment efficacy and overall survival in mice receiving allogeneic adoptive cell transfer [35], inhibited metastasis in a mouse melanoma model [36], and suppressed migration in glioblastoma cells [37].

Bevacizumab is a pharmacological therapy known to improve the quality of life and performance of patients with glioblastoma [32]. Furthermore, bevacizumab therapy has been linked to lower glucocorticoid needs, which are used to treat brain edema in glioblastoma patients but can have major adverse effects and cause significant morbidity [38]. Bevacizumab is an approved anti-angiogenic agent in glioblastoma and continues to be included in current treatment regimens [11]. Efforts to further optimize glioblastoma treatment approaches are ongoing [39].

The results of this study demonstrate the antiproliferative, cytotoxic, proapoptotic, and antimetastatic effects of the bevacizumab–fingolimod combination. These findings provide valuable preliminary data for designing alternative agent combinations and therapeutic strategies for glioblastoma, pending further pharmacological studies and in vivo analyses.

While analyses were performed on all culture samples, certain limitations of this study should be noted. Commercial cell lines consist of a single cell type, lacking the complex coordination mechanisms present in the human body. Consequently, experimental results from cell lines may not fully replicate the genotypic or phenotypic characteristics of human tissues, potentially leading to misleading conclusions [40]. This represents the first limitation of this research. Additionally, because this study utilized an in vitro experimental design, clinically relevant results can only be predicted. Unlike in vivo experiments, the absence of compensatory biological mechanisms limits the ability to draw direct conclusions about therapeutic outcomes. To achieve evidence closer to clinical applicability, the same mechanisms must be tested in vivo [41]. This constitutes the second limitation of the research. Despite these constraints, we believe that the findings of this research offer meaningful contributions to the future treatment of glioblastoma, paving the way for new therapeutic possibilities.

## 5. Conclusions

This study strongly demonstrated that the bevacizumab–fingolimod combination exhibits significant anticancer effects on C6 glioblastoma cells and can potentially reduce the aggressive and invasive characteristics of glioblastoma, indicating that it may offer a possible alternative and effective treatment option for glioblastoma. These promising findings particularly showed the combination’s capacity to decrease cell viability, trigger apoptosis, and inhibit migration. However, it is important to acknowledge that these early-stage findings were obtained from a single cancer cell line model and therefore require careful interpretation in light of limited experimental evidence. Critically, this study did not evaluate toxicity on normal cells or systemic effects, nor did it determine the combination’s ability to cross the blood–brain barrier and achieve therapeutic concentrations in a clinical setting, all of which are vital for any potential therapy. Therefore, while the initial results are encouraging, comprehensive future research is imperative to address these significant limitations. Such research must include toxicity profiling, in vivo efficacy studies in diverse preclinical glioblastoma models, and assessment of blood–brain barrier penetration. Further investigation into pharmacokinetic interactions and pharmaceutical properties is also necessary to fully explore its potential as an effective and safe treatment for glioblastoma.

## Figures and Tables

**Table 1:** Drug concentrations administered to the samples and the contents of the groups.

Groups	Contents	Final Concentration
Group 1 (Control)	None	-
Group 2	Treated with bevacizumab	0.06 µM
Group 3	Treated with fingolimod	8.27 µM
Group 4	Treated with bevacizumab + fingolimod	0.06 µM + 8.27 µM

The IC_50_ doses of the drugs applied to the cell lines were determined based on the MTT assay results from this study.

## Data Availability

The datasets generated and/or analyzed during the current study are not publicly available due to our possible patent application, but they are available from the corresponding author upon reasonable request, with the consent of all authors.

## Abbreviations

BBB	Blood–brain barrier
EGFR	Epidermal growth factor receptor
GBM	Glioblastoma grade IV
MAPK	Mitogen-activated protein kinase
mTOR	Mammalian target of rapamycin
PD-L1	Programmed death ligand-1
VEGF	Vascular endothelial growth factor

## References

[1] Sahoo L., Tripathy N.S., Dilnawaz F. (2024). Naringenin nanoformulations for neurodegenerative diseases. Curr. Pharm. Biotechnol..

[2] Mohammed S., Dinesan M., Ajayakumar T. (2022). Survival and quality of life analysis in glioblastoma multiforme with adjuvant chemoradiotherapy: A retrospective study. Rep. Pract. Oncol. Radiother..

[3] Oronsky B., Reid T.R., Oronsky A., Sandhu N., Knox S.J. (2021). A review of newly diagnosed glioblastoma. Front. Oncol..

[4] de Souza R.M., Shaweis H., Han C., Sivasubramaniam V., Brazil L., Beaney R., Sadler G., Al-Sarraj S., Hampton T., Logan J. (2016). Has the survival of patients with glioblastoma changed over the years?. Br. J. Cancer.

[5] Sabouri M., Dogonchi A.F., Shafiei M., Tehrani D.S. (2024). Survival rate of patient with glioblastoma: A population-based study. Egypt J. Neurosurg..

[6] Burnet N.G., Jefferies S.J., Benson R.J., Hunt D.P., Treasure F.P. (2005). Years of life lost (YLL) from cancer is an important measure of population burden--and should be considered when allocating research funds. Br. J. Cancer.

[7] Grech N., Dalli T., Mizzi S., Meilak L., Calleja N., Zrinzo A. (2020). Rising incidence of glioblastoma multiforme in a well-defined population. Cureus.

[8] Rios S.A., Oyervides S., Uribe D., Reyes A.M., Fanniel V., Vazquez J., Keniry M. (2024). Emerging therapies for glioblastoma. Cancers.

[9] Ferrara N., Hillan K.J., Gerber H.P., Novotny W. (2004). Discovery and development of bevacizumab, an anti-VEGF antibody for treating cancer. Nat. Rev. Drug Discov..

[10] Ferrara N., Gerber H.P., LeCouter J. (2003). The biology of VEGF and its receptors. Nat. Med..

[11] Garcia J., Hurwitz H.I., Sandler A.B., Miles D., Coleman R.L., Deurloo R., Chinot O.L. (2020). Bevacizumab (Avastin^®^) in cancer treatment: A review of 15 years of clinical experience and future outlook. Cancer Treat. Rev..

[12] Fujita T., Inoue K., Yamamoto S., Ikumoto T., Sasaki S., Toyama R., Chiba K., Hoshino Y., Okumoto T. (1994). Fungal metabolites. Part 11. A potent immunosuppressive activity found in Isaria sinclairii metabolite. J. Antibiot..

[13] Zhang L., Wang H. (2017). FTY720 inhibits the Nrf2/ARE pathway in human glioblastoma cell lines and sensitizes glioblastoma cells to temozolomide. Pharmacol. Rep..

[14] Chiba K., Kataoka H., Seki N., Shimano K., Koyama M., Fukunari A., Sugahara K., Sugita T. (2011). Fingolimod (FTY720), sphingosine 1-phosphate receptor modulator, shows superior efficacy as compared with interferon-β in mouse experimental autoimmune encephalomyelitis. Int. Immunopharmacol..

[15] Singh I.N., Hall E.D. (2008). Multifaceted roles of sphingosine-1-phosphate: How does this bioactive sphingolipid fit with acute neurological injury?. J. Neurosci. Res..

[16] Aslan J.E., You H., Williamson D.M., Endig J., Youker R.T., Thomas L., Shu H., Du Y., Milewski R.L., Brush M.H. (2009). Akt and 14-3-3 control a PACS-2 homeostatic switch that integrates membrane traffic with TRAIL-induced apoptosis. Mol. Cell.

[17] Wang X., Decker C.C., Zechner L., Krstin S., Wink M. (2019). In vitro wound healing of tumor cells: Inhibition of cell migration by selected cytotoxic alkaloids. BMC Pharmacol. Toxicol..

[18] Li J.H., Li S.Y., Shen M.X., Qiu R.Z., Fan H.W., Li Y.B. (2021). Anti-tumor effects of Solanum nigrum L. extraction on C6 high-grade glioma. J. Ethnopharmacol..

[19] Mosmann T. (1983). Rapid colorimetric assay for cellular growth and survival: Application to proliferation and cytotoxicity assays. J. Immunol. Methods.

[20] Budman D.R., Calabro A. (2002). In vitro search for synergy and antagonism: Evaluation of docetaxel combinations in breast cancer cell lines. Breast Cancer Res. Treat.

[21] Celik A., Bakar-Ates F. (2023). Alpha-lipoic acid induced apoptosis of PC3 prostate cancer cells through an alteration on mitochondrial membrane depolarization and MMP-9 mRNA expression. Med. Oncol..

[22] Du Z., Jia D., Liu S., Wang F., Li G., Zhang Y., Cao X., Ling E.A., Hao A. (2009). Oct4 is expressed in human gliomas and promotes colony formation in glioma cells. Glia.

[23] Vejselova Sezer C. (2024). Escin induces cell death in human skin melanoma cells through apoptotic mechanisms. Toxicol. Res..

[24] Spallotta F., Illi B. (2024). The role of HDAC6 in glioblastoma multiforme: A new avenue to therapeutic interventions?. Biomedicines.

[25] Zhang Y., Fang Z., Liu Z., Xi K., Zhang Y., Zhao D., Feng F., Geng H., Liu M., Lou J. (2024). Implantable microneedle-mediated eradication of postoperative tumor foci mitigates glioblastoma relapse. Adv. Mater..

[26] Laverty D.J., Gupta S.K., Bradshaw G.A., Hunter A.S., Carlson B.L., Calmo N.M., Chen J., Tian S., Sarkaria J.N., Nagel Z.D. (2024). ATM inhibition exploits checkpoint defects and ATM-dependent double strand break repair in TP53-mutant glioblastoma. Nat. Commun..

[27] Zhang J.F., Okai B., Iovoli A., Goulenko V., Attwood K., Lim J., Hess R.M., Abad A.P., Prasad D., Fenstermaker R.A. (2024). Bevacizumab and gamma knife radiosurgery for first-recurrence glioblastoma. J. Neurooncol..

[28] Pournajaf S., Afsordeh N., Bayat H., Pourgholami M.H. (2025). Fingolimod inhibits C6 rat glioma proliferation and migration, induces sub-G1 cell cycle arrest, mitochondrial and extrinsic apoptosis in vitro and reduces tumour growth in vivo. Clin. Exp. Pharmacol. Physiol..

[29] Friedman H.S., Prados M.D., Wen P.Y., Mikkelsen T., Schiff D., Abrey L.E., Yung W.K., Paleologos N., Nicholas M.K., Jensen R. (2009). Bevacizumab alone and in combination with irinotecan in recurrent glioblastoma. J. Clin. Oncol..

[30] Wick W., Gorlia T., Bendszus M., Taphoorn M., Sahm F., Harting I., Brandes A.A., Taal W., Domont J., Idbaih A. (2017). Lomustine and bevacizumab in progressive glioblastoma. N. Engl. J. Med..

[31] Johnson D.R., Leeper H.E., Uhm J.H. (2013). Glioblastoma survival in the United States improved after Food and Drug Administration approval of bevacizumab: A population-based analysis. Cancer.

[32] Johnson D.R., Omuro A.M.P., Ravelo A., Sommer N., Guerin A., Ionescu-Ittu R., Shi S., Macalalad A., Uhm J.H. (2018). Overall survival in patients with glioblastoma before and after bevacizumab approval. Curr. Med. Res. Opin..

[33] Chinot O.L., Wick W., Mason W., Henriksson R., Saran F., Nishikawa R., Carpentier A.F., Hoang-Xuan K., Kavan P., Cernea D. (2014). Bevacizumab plus radiotherapy-temozolomide for newly diagnosed glioblastoma. N. Engl. J. Med..

[34] Gilbert M.R., Dignam J.J., Armstrong T.S., Wefel J.S., Blumenthal D.T., Vogelbaum M.A., Colman H., Chakravarti A., Pugh S., Won M. (2014). A randomized trial of bevacizumab for newly diagnosed glioblastoma. N. Engl. J. Med..

[35] Marcus A., Eshhar Z. (2014). Allogeneic chimeric antigen receptor-modified cells for adoptive cell therapy of cancer. Expert Opin. Biol. Ther..

[36] LaMontagne K., Littlewood-Evans A., Schnell C., O’Reilly T., Wyder L., Sanchez T., Probst B., Butler J., Wood A., Liau G. (2006). Antagonism of sphingosine-1-phosphate receptors by FTY720 inhibits angiogenesis and tumor vascularization. Cancer Res..

[37] Zhang L., Wang H., Zhu J., Ding K., Xu J. (2014). FTY720 reduces migration and invasion of human glioblastoma cell lines via inhibiting the PI3K/AKT/mTOR/p70S6K signaling pathway. Tumour Biol..

[38] Touat M., Idbaih A., Sanson M., Ligon K.L. (2017). Glioblastoma targeted therapy: Updated approaches from recent biological insights. Ann. Oncol..

[39] Kim M.M., Umemura Y., Leung D. (2018). Bevacizumab and glioblastoma: Past, present, and future directions. Cancer J..

[40] Yilmaz I., Karaarslan N. (2022). Examining the effects of HMG-CoA reductase inhibitors on anabolic and catabolic signaling pathway proteins associated with degenerative disc disease. Eur. Rev. Med. Pharmacol. Sci..

[41] Yilmaz I., Akalan H., Yasar Sirin D., Karaarslan N., Kaplan N., Ozbek H. (2022). Effects of an acetylcholinesterase inhibitor and an N-methyl-D-aspartate receptor antagonist on inflammation and degeneration of the nucleus pulposus. Eur. Rev. Med. Pharmacol. Sci..

